# Review: Engineering of thermostable enzymes for industrial applications

**DOI:** 10.1063/1.4997367

**Published:** 2018-01-11

**Authors:** Federica Rigoldi, Stefano Donini, Alberto Redaelli, Emilio Parisini, Alfonso Gautieri

**Affiliations:** 1 Biomolecular Engineering Lab, Dipartimento di Elettronica, Informazione e Bioingegneria, Politecnico di Milano, Piazza Leonardo da Vinci 32, 20133 Milano, Italy; 2 Center for Nano Science and Technology at Polimi, Istituto Italiano di Tecnologia, Via G. Pascoli 70/3, 20133 Milano, Italy

## Abstract

The catalytic properties of some selected enzymes have long been exploited to carry out efficient and cost-effective bioconversions in a multitude of research and industrial sectors, such as food, health, cosmetics, agriculture, chemistry, energy, and others. Nonetheless, for several applications, naturally occurring enzymes are not considered to be viable options owing to their limited stability in the required working conditions. Over the years, the quest for novel enzymes with actual potential for biotechnological applications has involved various complementary approaches such as mining enzyme variants from organisms living in extreme conditions (extremophiles), mimicking evolution in the laboratory to develop more stable enzyme variants, and more recently, using rational, computer-assisted enzyme engineering strategies. In this review, we provide an overview of the most relevant enzymes that are used for industrial applications and we discuss the strategies that are adopted to enhance enzyme stability and/or activity, along with some of the most relevant achievements. In all living species, many different enzymes catalyze fundamental chemical reactions with high substrate specificity and rate enhancements. Besides specificity, enzymes also possess many other favorable properties, such as, for instance, cost-effectiveness, good stability under mild pH and temperature conditions, generally low toxicity levels, and ease of termination of activity. As efficient natural biocatalysts, enzymes provide great opportunities to carry out important chemical reactions in several research and industrial settings, ranging from food to pharmaceutical, cosmetic, agricultural, and other crucial economic sectors.

## I. THERMOSTABLE ENZYMES IN INDUSTRIAL APPLICATIONS

Enzymes have long been used in detergents and for food production, especially cheese, sourdough, beer, and wine as well as for the production of leather, indigo, and linen.^1,2^ Such processes are usually triggered by enzymes produced by specific microorganisms or by enzymes that are present in natural products, such as, for instance, the papaya fruit, hence, not used as pure isolated molecules. The development of fermentation processes has allowed the large-scale production, isolation, and purification of enzymes from selected production strains, thus making it possible to provide biocatalysts to be used as molecular tools in actual industrial processes, such as, for instance, in detergent, textile, and starch industries. In particular, a commercial breakthrough occurred in 1957 with the first commercial protease production by Novozymes.^1^

More recent advancements in protein engineering and directed evolution have made the continuous development of new and more efficient enzymes a reality. Indeed, mutant enzymes for established technical applications or new tailor-made enzymes for areas of application where enzymes had not been previously used have been successfully introduced. Of those enzymes that are used in industrial processes, over half are from fungi, over one-third is from bacteria, and the rest originate from animal (8%) and plant (4%) sources.^3^ Recombinant DNA techniques have allowed the isolation and cloning of genes encoding for enzymes from all possible sources, including microbes and other microorganisms that are particularly difficult to manipulate, and high-yield heterologous protein expression. As a result, this convenient technology has increased production levels and has shifted enzyme production from bacterial strains that are not suited for industry into industry-friendly microorganisms such as *Aspergillus*, *Trichoderma*, *Kluyveromyces*, *Saccharomyces*, *Yarrowia*, and *Bacillus.*^4^

On a large scale, the enzymes produced by microbial strains are economical due to high production levels associated with standard expression, ease of growth, inexpensive culture media, and short fermentation cycles. Downstream processing allows for a rapid examination of thousands of cultures. Furthermore, different microbes produce somewhat different enzymes that catalyze the same reaction, providing high levels of flexibility.

The International Union of Biochemistry (IUB) categorizes enzymes in six different classes, based on the enzyme action mechanism. The six enzyme classes are ligases, isomerases, oxidoreductases, lyases, transferases, and hydrolases. Currently, more than 75% of the enzymes that are used commercially are members of the hydrolase family and are employed for the degradation of a number of different natural substances. Of all the commercial hydrolases, proteases are the major and most important sub-type. Indeed, they are widely used in both the detergent industry and the starch industry. Proteases are also important components in textile, animal feed, and dairy industries. The second largest sub-group is represented by carbohydrases, mostly amylases and cellulases. For instance, carbohydrases are extensively used in productive sectors such as starch, textile, detergent, and baking industries, which is where most industrial enzymes are used.

It has been estimated that by 2018, the global market for industrial enzymes will surpass the USD 7.1 billion mark and its five-year compound annual growth rate (CAGR) will be around 8.2%. The market for food enzymes alone is projected to reach USD 2.94 billion by 2021, at a CAGR of 7.4% between 2016 and 2021. Moreover, it is expected that the maximum growth rate will be observed in the detergent enzyme segment (CAGR of 11.3% in the 2016–2021 period).^5^ Proteases were the prominent product segment in 2015, accounting for 27.4% of the global enzyme market; now, they are expected to show an even more profitable growth in light of their increasing application in pharmaceutical, detergent, and chemical sectors.^6^ The most relevant industrial applications of enzymes are summarized in Table I.

**TABLE I.** The most relevant industrial applications of bacterial/fungal enzymes: in the first column, the enzyme name is specified, followed by its International Union of Biochemistry (IUB) class, the typical substrate, and its main applications. The IUB classes are Oxido-Reductases (1), Transferases (2), Hydrolase (3), Lyases (4), Isomerases (5), and Ligases (6).

Enzyme	IUB classes	Substrate classes	Industry field (application)
α-amylase (or glycogenase)	3	Carbohydrates	Baking (softness and volume of bread), laundry and detergents (starch strain removal), paper and pulp (deinking, drainage improvement, and starch coating), starch and fuel (starch liquefaction and saccharification), textile (removal of starch from woven fabrics and de-sizing), and food (juice treatments, low calorie beer, glucose, and fructose syrup production)
α-galactosidase	3	Glycolipids and glycoproteins	Dairy
β-glucanase	3	Glucose polysaccharide	Animal feed (digestibility improvement) and food (mashing)
Acetolactate-decarboxylase	4	(S)-2-hydroxy-2-methyl-3-oxobutanoate (C-C bond)	Food (beer maturation)
Acylase	3	Penicillin	Organic synthesis (synthesis of semisynthetic penicillin)
Amyloglucosidase	3	Maltooligo- and polysaccharides	Personal care (antimicrobial combined with glucose oxidase) and starch and fuel (saccharification)
Asparaginase	3	Asparagine	Pharmaceutical (treatment of acute leukemia) and food (decrease acrylamide)
Catalase	1	Hydrogen peroxide	Food and textile (bleach termination)
Cellulase	3	Cellulose and related polysaccharides	Plant waste treatment, paper and pulp (modification of fibers, deinking, and drainage), textile (denim finishing and softening of cotton), cleaning (removal of stains), color clarification, and anti-redeposition
Cyclodextrin-glycosyltransferase	2	Cyclic dextrins (starting from polysaccharides)	Cyclodextrin production
Dextranase	3	Dextran	Pharmaceutical and food
Glucose isomerase	5	d-glucose and d-xylose	Starch and fuel and fructose syrup (glucose to fructose conversion)
Glucose oxidase	1	Glucose	Baking (strengthening of dough) and personal care (bleaching and antimicrobial)
Invertase	3	Sucrose	Food
Lactase	3	Lactose	Food (juice clarification, beer flavor, cork stopper treatment, and milk lactose removal) and textile (bleaching)
Lipase	3	Lipids	Baking (stability of dough and conditioning), food (cheese flavoring), laundry and detergents (lipid stain removal), textile (de-inking, de-pickling, and cleaning), organic synthesis (resolution of chiral alcohols and amides), fats and oils (transesterification and de-gumming, lysolecithin production for phospholipases), and paper and pulp (pitch control and contaminant control)
Lipoxygenase	1	Polyunsaturated fatty acids	Baking (bread whitening and dough strengthening)
Mannase	3	β-mannose, galactomannan, and galactose	Laundry and detergent (reappearing stain removal) and animal feed additive
Naringinase	3	Naringin (flavanone-7-O-glycoside)	Food
Nitrilase	3	Nitriles	Organic synthesis (synthesis of enantiopure carboxylic acids)
Oxidoreductases	1	Various compounds: catalyzing the removal of hydrogen atoms and electrons	Food and detergents
Pectinase	3	Pectin	Food (clarification, mashing, and de-pectinization of fruit-based products) and textile (scouring)
Pectin methyl esterase	3	Pectin	Food (firming fruit-based products)
Penicillin amidase	3	Penicillin	Pharmaceutical
Phytase	3	Phytic acid	Animal feed (phytate digestibility-phosphorous release)
Peroxidase	1	Hydrogen peroxide and hydroperoxides	Textile (removal of excess dye) and personal care (antimicrobial)
Protease	3	Protein and peptide	Laundry and detergents (protein stain removal), baking, food (cheese making, milk clotting, low allergenic infant products, flavor, and brewing for clarification-low calorie beer), leather (de-hiding), pharmaceutical (treatment of blood clot), textile (unhearing and bating), paper and pulp (biofilm removal), and fuel (yeast nutrition)
Pullulanase	3	Polysaccharide	Baking, Starch, and Fuel (saccharification)
Rennin	3	Protein (k-casein)	Food (precipitation and curd formation in cheese-making)
Subtilisin	3	Protein and peptide	Pharmaceutical, laundry, and detergents
Transglutaminase	2	Protein and peptide	Baking (laminated dough strengths) and food (modification of visco-elastic properties)
Xylanase	3	Polysaccharide β-1,4-xylan	Baking (conditioning of dough), paper and pulp (bleach boosting), animal feed (digestibility improvement), and starch and fuel (viscosity reduction)

At the industrial level, enzymes are mostly used as detergent additives. Indeed, detergents are supplemented with proteases, lipases, amylases, oxidases, peroxidases, and cellulases in order to breakdown different types of chemical bonds in water. To this end, it is essential that they maintain their activity at high temperatures (60 °C) and high pH values (pH 9–11), in particular when mixed with other washing powder components. Nearly 25% of the total worldwide sales of enzymes is represented by proteases that are added to laundry detergents. In Secs. I A–I H, the most important families of industrially relevant enzymes are described, together with key applications.

### A. Pectinases

In paper and textile industries, enzymes are increasingly utilized not only to develop cleaner processes but also to reduce both raw material usage and waste production. For instance, an enzymatic process based on a pectate lyase that allows the low-temperature removal of pectin and other hydrophobic materials from cotton fabrics has been developed.^7^ The food industry also takes advantage of pectinases, particularly for the clarification of fruit juices, for the degumming of fibers, and for wine making.

### B. Cellulases

Cellulose is a renewable resource with great potential for bioconversion to value-added bioproducts. Cellulose can be degraded by cellulases produced by cellulolytic bacteria. Cellulases are among some of the most important industrial enzymes known to date.^8^ For instance, they convert cellulose to sugars that are suitable for human consumption. On a large scale, these sugars can in turn be fermented to generate bioethanol and biobased products.^8^ Cellulases also find application in the textile industry, where they are used for the polishing of fabrics, and in the laundry detergent industry.^9^ A cellulase from *Streptomyces thermoviolaceus* with high thermal and pH stabilities has been shown to be more active than other commercial cellulases in the presence of detergents.^10^

### C. Xylanases

Xylanases play a key role in the enzymatic depolymerization of hemicellulose to yield monomeric sugars. Traditionally, these enzymes are used in food and paper industries. In recent years, they have received growing attention for the production of sugars from lignocelluloses. Xylanases are also used for the bleaching of rice straw pulp.^11^ Xylanases from *Actinomadura* sp. *FC7* and *Nonomuraea flexuosa* have been shown to have high thermostability.^12,13^ Owing to their high thermal and pH stabilities, fused xylanases from fungi and actinomycetes have been used in paper and pulp industries.^14^ *Streptomyces* spp. are able to produce high levels of xylanase and provide efficient biobleaching.^11^ Similarly, they are able to hydrolyze straw waste and produce biogas.^15^

### D. Amylases

Common applications for this family of enzymes are in bakery, brewing, and alcohol industries, where thermophilic and acidophilic amylases from *Streptomyces erumpens* are utilized.^16,17^ Thermostable amylases from *Nocardiopsis* sp. are also used in bakery and paper industries.^18^ The amylase from *Thermobifida* sp. is used for the production of maltotriose from starch.^19^ Moreover, in the starch processing industry, a number of other end product-specific amylases are commonly used for the synthesis of different maltooligosaccharides.^20^ Finally, several actinomycetes are the source of cold-active *α*-amylases that find application in textile, detergent, and bioethanol industries.^21^

### E. Proteases

Proteases are utilized in the dairy industry for the manufacturing of cheese. Due to its high specificity, calf rennin has generally been the protease of choice in cheese-making. However, rennin is gradually giving way to microbial proteases from microorganisms such as *Mucor miehei*, *Bacillus subtilis*, *Endothia parasitica*, and *Aspergillus oryzae MTCC 5341*. Among proteases, aminopeptidase hydrolyzes amino acid residues from the N-terminal portions of proteins. Aminopeptidases have a wide range of applications in various fields such as the pharmaceutical industry, where they represent an important molecular tool for protein sequence analysis,^22^ and the food industry, for flavor enhancement.^23,27^ When combined with other proteases, they lead to a complete degradation of proteins, such as casein, gluten, collagen, and gelatin, helping in nutrient utilization.^24^

### F. Lipases

Lipases hydrolyze long chain triglycerides to form diglycerides, monoglycerides, fatty acids, and glycerol.^25,31^ Besides their ability to hydrolyze carboxylic ester bonds, lipases can catalyze esterification reactions in non-aqueous media. Lipases find application in food, detergent, pharmaceutical, leather, textile, cosmetic, and paper industries.^26,27^ In the food industry, lipases are used for fat and oil processing. Interestingly, lipases from different organisms provide different positional specificities, fatty acid specificities, thermal stabilities, and optimum pH values.^26^ Detergent formulations also include lipases, which are of great help for the removal of lipid stains, fatty food stains, and sebum from fabrics. Alkaline yeast lipases can work at lower temperatures than bacterial and mold lipases. Cold-active lipases are used as components of detergents for cold washing, with clear advantages in both energy consumption and textile durability.

### G. Laccases

Laccases are blue multicopper oxidases that participate in the degradation of polymers and ring cleavage of aromatic compounds. Owing to their ability to oxidize lignin-related compounds and highly resistant environmental pollutants, they are used in several biotechnological processes such as for instance wastewater treatment and detoxification. Their typical substrates are amines and phenols. These enzymes are also used as medical diagnostic tools and biosensors, in biofuel cells, for the bioremediation of herbicide- and pesticide-contaminated soil, as cleaning agents in water purification systems, as catalysts in drug manufacturing, and as ingredients in cosmetics.^28,29^

### H. Phytases

Phytases are used both as an animal feed ingredient and in foods to improve plant phosphorus uptake by animals.^30^ Phytases allow the release of phosphorus from plant feedstuffs, where about 2/3 of phosphorus is stored as phytate. Hydrolysis of phytate blocks the translocation of phosphorous into the soil, where it causes eutrophication. In the food industry, phytases are utilized to remove phytic acid. These enzymes are found in many bacteria, yeasts and fungi. New fungal phytases showing high specificity or thermostability have also been identified.^31^

## II. THERMOSTABLE ENZYMES IN NATURE

### A. Extremozymes

Thermophiles are organisms that have evolved to strive in extreme conditions such as temperatures ranging up to 120 °C, high pressure values (up to 250 atm), or extreme pH or salt conditions (up to 5% of NaCl).^32^ Their cellular components are also thermostable, including their enzymes, sometimes referred to as extremozymes, which are known to withstand high temperature and extremely acidic and alkaline conditions, and they generally exhibit increased resistance to denaturation^33^ and proteolysis.^34^ Thermophiles have long been considered of high industrial importance for their possible use in many technological processes, either as intact organisms or as a source of thermostable enzymes that can catalyze specific reactions at high temperatures. However, the majority of enzymes that are currently used in industry are obtained from fungi or mesophilic bacteria. To date, only a few extremozymes have been used for industrial applications, mostly involving DNA polymerase. Today, beside their use in DNA replication, new challenges have broadened their range of successful utilization. One of the major problems in the use of functional extremozymes is the establishment of proper and fine-regulated production conditions such as hosts, efficient transformation approaches, and adequate expression vectors. Due to differences in codon usage with respect to commonly used expression systems such as *Escherichia coli* or *Bacillus* sp., only a few systems have been successfully utilized, mostly members of the genus *Thermus* or the hyperthermophilic species *Sulfolobus solfataricus.*^35,36^

### B. Thermophiles used in industry

There is hardly any example that can represent the impact of thermophiles in all aspects of our daily life better than the thermostable DNA polymerases that are used in the polymerase chain reaction (PCR). Nearly two and a half decades after the Nobel prize in chemistry given to Mullis and Smith in recognition of their pioneering work on the development of the technique, biomedical and biotechnology research have advanced far beyond levels that would have been unimaginable without it. Thermophilic organisms such as *Thermus aquaticus, Pyrococcus furiosus, and Thermococcus litoralis* have provided those stable and proofreading forms of DNA polymerases (usually referred to as Taq, Pfu, and Vent) that survive beyond the denaturation temperature of long DNA fragments and, hence, have allowed the technique to become routinely and efficiently used in all the molecular biology laboratories around the world.

A further example of the importance of extremophilic enzymes for industrial applications can be found in the starch field. The standard process for starch conversion into single glucose units occurs in two steps: (1) liquefaction of the raw starch granules followed by (2) saccharification.^37^ The liquefaction of the raw starch granules is achieved through a necessary heating step (105** °**C for 5 min and 95** °**C for 1 h at pH 6.0) to facilitate liquefaction, and then, saccharification is done at 60** °**C for 3 h at pH 4.5.^38^ Currently, the key enzymes that are used for the production of glucose from starch are typically a bacterial amylase and a fungal glucoamylase combined with a pullulanase.^39^ Since these enzymes are not active at high temperatures and low pH values (as needed in the second step of the process), cooling and pH adjustment is mandatory. This energy and time consuming procedure has been optimized by the use of more suitable extremophilic amylolytic enzymes.^40^ The first archaeal amylase with an optimum temperature of about 100** °**C and residual activity at 130** °**C was found in *P. furiosus*, and it was characterized in 1990.^41^ Recently, an acid-stable amylase with a half-life of 30 min at 80** °**C was described.^42^ To date, one of the most heat-active pullulanase, having an optimum temperature of 100** °**C, has been discovered in *Thermococcus kodakarensis KOD1.*^43^ One of the most heat-active starch-degrading enzymes known to date is an α-amylase from *Methanococcus jannaschii* (optimum temperature = 120** °**C).^44^

Examples of the most common enzymes that exist in thermophilic microorganisms and that, due to their higher thermal stability compared to their mesophilic homologues, are commonly used in high temperature biotechnology processes include cellulases, amylases, xylanases, lipases, proteases, pectinases, and esterases. In recent years, the use of extremophiles as cellular biocatalysts for biotransformation, in particular for biofuel production, has attracted growing interest owing to the accelerated reaction rates, the reduced energy input, and the low contamination risk that are associated with the process. Moreover, their ability to exploit different carbohydrate sources (such as starch or hemicellulose) further favors the use of extremophilic species in industrial bioprocesses. In this field, the current main focus is on the use of starch- and lignocellulose-degrading enzymes for the production of next-generation biofuels.

### C. Nature's strategies to achieve thermostability

Several attempts to identify those key natural features, either at the sequence or at the structural level, that provide all proteins with their signature thermostability profile have failed to paint a clear picture and to define first principles of universal validity and general applicability. At most, consistent trends have been described when comparing different members of specific protein families, where a medium-to-high degree of sequence and structure similarity is often observed. In general, thermophilic proteins are mostly made of both hydrophobic and charged residues, showing a smaller proportion of uncharged polar residues compared to mesophilic proteins. Hydrophobic residues, which usually cluster in the core of the protein to minimize solvent exposure, form stabilizing van der Waals interactions with other hydrophobic residues, possibly exploiting the lack of directionality that is associated with these contacts and therefore adopting the conformation that minimizes voids in the structure while maximizing the contact surface area. Accordingly, thermophilic proteins usually have a higher than average content of valine (Val) and isoleucine (Ile) residues compared to mesophilic proteins.^45,46^ On the other hand, properly oriented charged residues can allow the formation of stabilizing salt bridges, whose energy contribution to the stabilization of the protein fold often exceeds that of hydrophobic interactions. The stabilization energy provided by a single salt bridge is usually estimated to be in the order of 3–5 kcal/mol. The need for a proper orientation of charged residues to form stabilizing electrostatic interactions and, at the same time, to avoid the clustering of identical charges highlights the limitations that are intrinsic in any thermostability analysis approach that focuses only on residue counts and distribution within primary sequences without considering structure level comparisons between proteins. As previously mentioned, the tendency of hydrophobic residues to cluster and shield themselves from the aqueous environment is one of the main driving forces behind the folding and the thermal stability of a protein. Yet, within the class of hydrophobic residues, further stabilization is often provided when aromatic interactions (such as π-π, cation-π, and S/π interactions) can be formed, suggesting a complex interplay between the size, chemical nature, and electronic structure of the hydrophobic residues involved. Disulfide bond formation resulting from two spatially closed cysteines is also an important driving force in protein folding. In fact, disulfide bridges are common in nature. The strength of the covalent bond that is formed between two oxidized cysteines clearly provides a great deal of energy stabilization to the fold of the protein, either locally or globally. Interestingly, Fitter and colleagues^47^ analyzed structural fluctuations of α-amylases from both mesophilic and thermophilic organisms, demonstrating that in the thermophilic protein, the folded state is more structurally flexible than the folded state of the mesophilic homologue. In contrast to the general rule that suggests that higher structural rigidity corresponds to higher thermostability,^48^ in this case, higher thermostability could be the result of entropic stabilization. Altogether, these different strategies adopted by extremophiles to achieve their superior stability can be used to guide the design and engineering of novel enzymes.

## III. ENZYME STABILIZATION THROUGH DIRECTED EVOLUTION

### A. Directed evolution

**FIG. 1.**
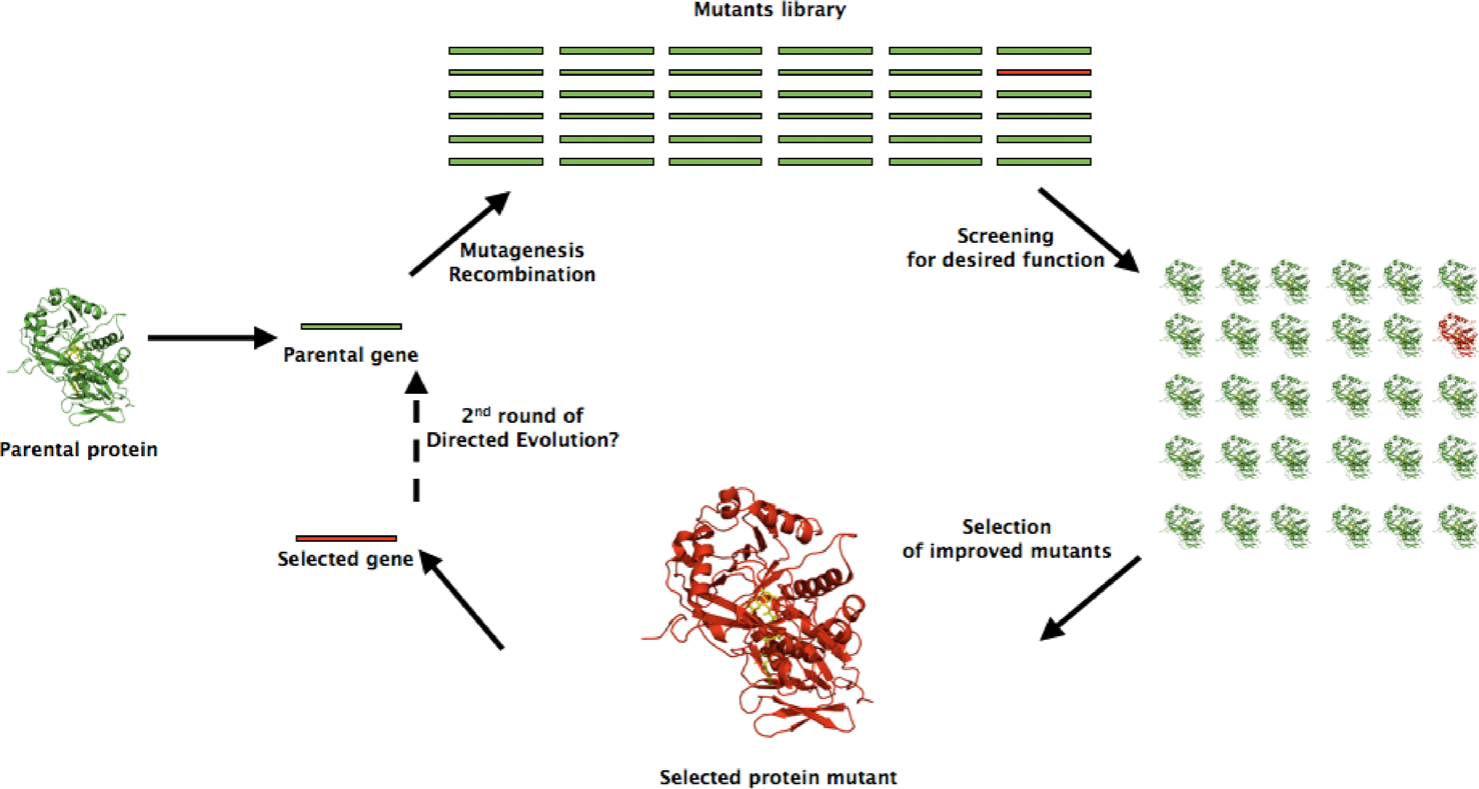
Directed evolution campaign. An iterative mutagenesis and screening process is performed starting from the gene coding for the enzyme of interest. From the generated mutant library, mutants are screened for the desired function or property. The best performing mutant can then be used as the parental gene for the next iterative rounds of mutagenesis.

Directed evolution has become a fundamental strategy in protein engineering for the production of more powerful and efficient biocatalysts.^49–54^ The process is similar to natural evolution, albeit on a reduced timescale. In fact, directed evolution generates, and selects under a specific evolutionary pressure, enzymes with novel or improved features through an iterative process characterized by several rounds of mutagenesis and screening, starting from a parental protein. Generally, the creation of random mutants from a parental protein is done by error prone mutagenesis,^55–57^ DNA shuffling,^58–60^ site-saturation mutagenesis,^61,62^ chemical mutagenesis,^63,64^ or using different mutator strains.^65–67^ However, all these methods generally require reiterative manipulation of single genes and are not used for parallel and continuous directed evolution of gene networks or genomes. In this regard, Wang, Isaacs, and colleagues developed the so-called Multiplex Automated Genome Engineering (MAGE) approach that, by coupling parallel DNA synthesis and recombination in a single *E. coli* cell or across a population of cells, leads to the generation of multiple modifications (mismatches, insertions, and deletions), from single point mutations to the genome level.^68,69^ Due to the large size of the resulting libraries, the ability to identify and isolate those mutants that feature the desired properties is a critical success factor in a directed evolution campaign. To face this burden, different strategies are adopted for library analysis and can be divided into two main methods: screening and selection. While the selection approach directly identifies the desired mutant, eliminating unproductive mutations based on a direct connection between cell growth and an optimized or acquired enzyme function, in a screening method, all the resulting variants from the library are individually tested for the desired function, minimizing the risk of false negatives. However, the weakness of this method lies in the reduced number of mutants that can be evaluated, which makes automation a necessary and key element for a rapid high-throughput screening approach. Microtitre plate- and agar plate-based screening procedures are the two most commonly adopted library screening formats. Compared to the latter, microtitre plate-based assays are suitable for handling large libraries of mutants and use a smaller quantity of the sample. Generally, an enzyme activity assay is performed on crude lysate from single colonies, or on purified proteins, into multi-well plates (e.g., from 96 to 9600-well). The use of classical colorimetric or fluorescence assays allows the verification of any small improvements in the desired function or property of the enzyme.^70–73^ However, the use of these assays is restrained only to those cases where substrates, cofactors, or products are suitable for UV-vis absorbance or fluorescence measurements. In order to overcome this limitation, a variety of analytical techniques can be implemented within a high-throughput format. Examples include automated systems using on-line high performance liquid chromatography (HPLC)^74^ or mass spectrometry (MC)^75^ for direct product quantitation. Agar plate-based screening methods focus on the direct correlation between the growth of the host organism on selective agar plates for the screening of a specific enzyme function.^56,76,77^ Here, colonies displaying a desired color within a certain time window are selected as active. Despite the outstanding performance of this approach for the direct detection of the desired mutant, its lower sensibility in the estimation of the enzymatic activity represents its main limitation compared to a microtitre plate-based screening method. Therefore, in order to identify significantly improved enzyme variants, it is common to couple an agar plate assay as a primary screen with a secondary activity assay, generally set up in a 96-well microtiter plate and based on a biochemical assay.^78^ Microtitre plate- and agar plate-based screens are powerful methods for library analysis; however, relative to the size of complete libraries (10^8^–10^9^ mutants), their efficiency is relatively low (∼10^4^/day for the microtitre plate and ∼10^5^/day for the agar plate). Interestingly, fluorescence-activated cell sorting (FACS) provides a high-throughput approach directly on a cell population (∼10^8^/day), for the direct separation of mutants expressing the desired protein, where the link between the genotype (mutation) and the phenotype (desired property) is maintained through a fluorescence assay that detects those cells that display that particular enzymatic activity.^79^ Different strategies can be adopted, generally based on the detection of the product either inside the cell (product entrapment)^80^ or onto the surface of cells (cell surface display).^81^ Both these methods can be classified as *in vivo* methods. A FACS-based *in vitro* approach, usually referred to as *in vitro* compartmentalization, involves compartmentalization of the gene encoding the mutant protein in small aqueous droplets, together with the fluorescent product of interest.^82^ In practice, a directed evolution campaign can be separated into three well-defined steps: the identification of a good starting parental protein, the creation of a library of mutants, and the screening or selection step, based on the artificial selection imposed to identify improved mutants that carry out a specific function. This entire process of mutant generation and isolation is repeated until the desired change is observed, and it can be iterated over the resulting mutant from a previous cycle until no further change is elicited (Fig. 1). It is generally believed that the use of a consensus sequence that is either based on a protein family of interest or closer to the protein ancestor will likely define the most stable starting scaffold to be used in a directed evolution campaign, and it will be more tolerant to the deleterious effects of mutations or insertions. Indeed, protein families that are characterized by substrate ambiguity (i.e., are active on a wider range of substrates) or catalytic promiscuity (i.e., catalyze different types of reactions) are more suitable for a directed evolution campaign.^83–88^ Clearly, directed evolution represents a powerful and effective approach for reshaping the basic characteristics of enzymes or for designing *de novo* enzymes in order to improve not only their catalytic features (e.g., optimization of kinetic parameters) but also their stability (e.g., thermal and pH stabilities) or protein yield production for subsequent industrial applications.

### B. Thermostable enzymes by directed evolution

Several examples of thermal stabilization obtained by directed evolution can be found in the literature. For instance, improvements in the thermostability of galacto-N-biose/lacto-N-biose I phosphorylase (GLNBP) from *Bifidobacterium longum* JM1217 were achieved by directed evolution.^89^ From the initial library of GLNBP mutants, two single mutants were selected, showing each a significant improvement relative to the wild type (∼10 °C). Based on these results, the corresponding double mutant was generated and shown to exhibit a 20 °C higher thermostability than the wild type, allowing its use for the industrial production of LNB at high temperature, thus resulting in the shortening of the reaction time and in the prevention of microbial contamination. A study reporting the enhancement in the thermostability of an amylase from *Thermus* sp. strain IM6501 (ThMA) well represents the compromises that can be faced in a directed evolution experiment, showing how an improvement in thermostability can affect catalytic efficiency.^90,91^ Indeed, the resulting thermostable mutant ThMA, which features a total of seven single mutations, exhibits a 15 °C increase in the optimal reaction temperature relative to the wild type enzyme. However, one of the mutations reduces the activity of the enzyme by 23% relative to the wild type form, still preserving significant thermoresistance.^92^ Another example of thermoresistance improvement by directed evolution for industrial applications comes from the endo-β-1,4-xylanase (XynA) from *Thermomyces lanuginosus.*^93^ Here, based on a first campaign of directed evolution, four mutants, which were selected based on the exhibited improvement of activity and stability, were subjected to further rounds of mutagenesis. The majority of the resultant mutants exhibited the expected compromise between stability and activity, and only one of these second generation mutants showed a significant improvement in activity and stability relative to the wild type enzyme. It is worth stressing that directed evolution experiments can target not only high thermostability improvements, as described above. For instance, aggregation can significantly influence the yield and the biological activity of biopharmaceutical products.^94^ For example, antibody stabilization is a critical issue for industry. Aggregation-resistant antibodies, showing a 2–3 °C increase compared to the wild type, have been developed by directed evolution in order to improve antigen-binding fragment (Fab) stability.^95^ Another example involving a combination of rational design and directed evolution is that of an extremely stable green fluorescent protein, eCGP123, which was created by a consensus engineering approach or consensus green protein (CGP).^96^ The process consisted of a recursive iteration involving the sequential introduction of three destabilizing heterologous inserts, a sequential mutagenesis step to overcome the destabilization, and the final removal of the destabilizing insert from the mutated gene.^97^

## IV. ENZYME STABILIZATION THROUGH RATIONAL DESIGN

The vast majority of mutations in a natural enzyme destabilize.^98^ Nonetheless, a naturally occurring enzyme is not necessarily the most stable form that is possible for that specific enzyme. Indeed, some mutations can further stabilize a protein, increasing the equilibrium population of the folded state.^83,99^ In a directed evolution campaign, whereby random mutations are introduced in the coding gene and the large resulting libraries (typically consisting of more than 10^5^ variants) are screened for a specific function, mutant enzymes featuring improved thermostability can most likely be identified.^100,101^ Unfortunately, such a random approach is possible only with enzymes for which fast activity screens can be implemented. While directed evolution has yielded improved enzyme variants, it is a time-consuming and highly labor-intensive method, often leading to a dead-end, where further introduction of function-altering mutations is limited by low enzyme stability.^102^

### A. Rational design

The modification of the properties of an enzyme could be achieved through the use of different rational approaches. Over the past few years, computational design has been successfully applied for the thermostabilization of noncatalytic proteins, the first notable examples being represented by the computational stabilization of a cytokine analog in 2002 by Luo and colleagues.^103^ However, the stabilization of an enzyme presents additional challenges. Indeed, the geometry of the active site and the protein dynamic behavior during an enzymatic reaction are often crucial for providing the maximum catalytic efficiency. Therefore, rational design methods need to be able to predict stabilizing mutations in the context of a given fold and, at the same time, minimize any change in the backbone conformation that might disrupt the structure of the active site or reduce its flexibility. Recently, several different *in silico* methods to establish the effect of mutations on the stability of a protein have been developed.^104,105^ Nonetheless, the reliability of these approaches is still unsatisfactory.^106^ The techniques used to increase protein stability with a rational approach are often based on one or multiple methods including phylogenetic analysis,^107^ comparison to homologous proteins (and particularly thermophiles),^108^ optimization of charged interactions (salt bridges and hydrogen bonds), optimization of residues and loops showing unfavorable Ramachandran angles and high B-factors,^109^ methods based on the calculation of free energies,^110^ and structure-based computational design.^111,112^

**FIG. 2.**
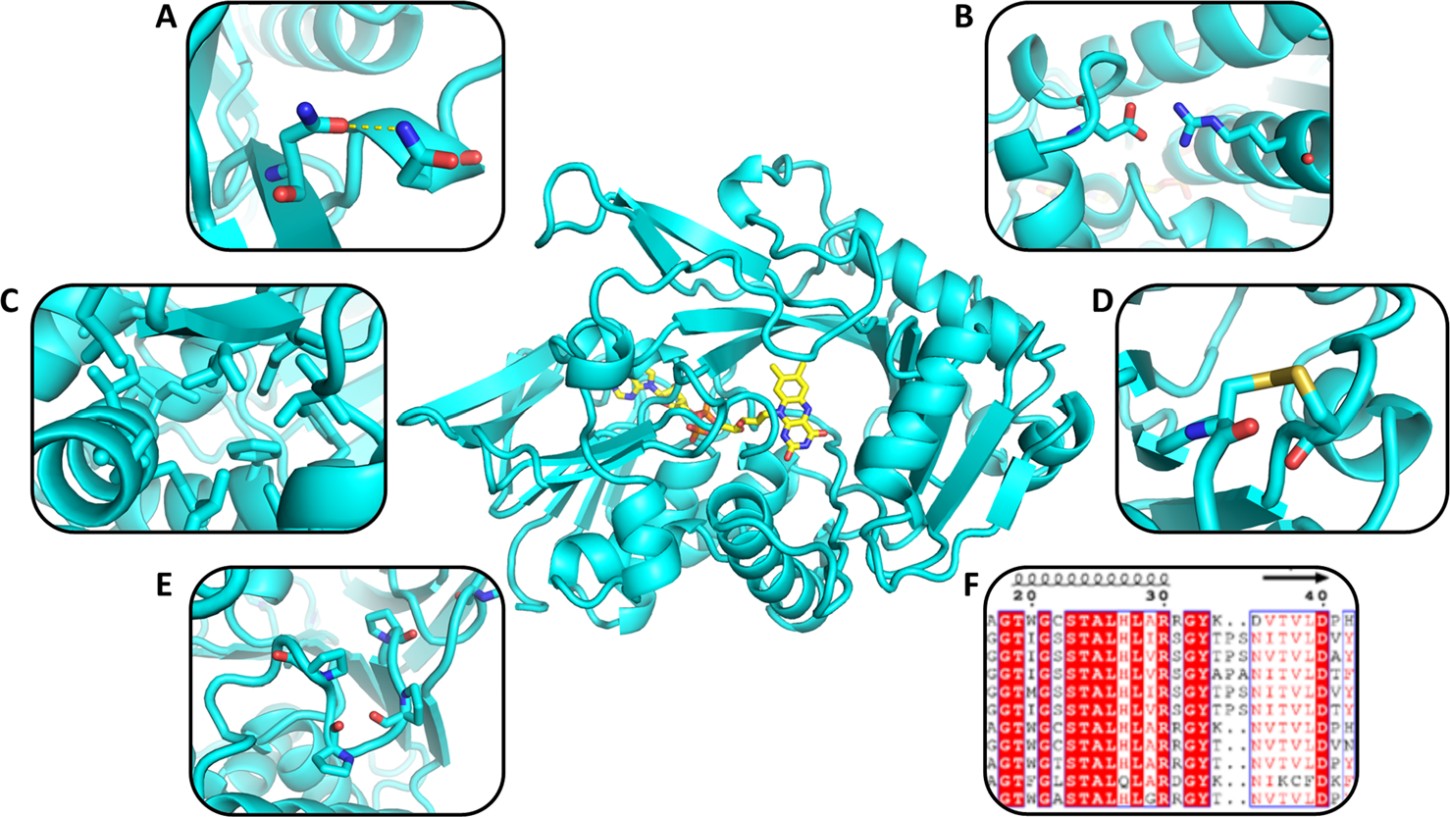
Stabilizing strategies used in rational enzyme design. The most common strategies involve the introduction of surface hydrogen bonds (a) and salt bridges (b), the stabilization of the hydrophobic core (c), the introduction of disulfide bridges (d), and the stabilization of mobile loops using prolines (e). Phylogenetic analysis (f) can be used alone or in combination with previous strategies to guide the enzyme rational design process.

Several rational design strategies can be implemented to improve enzyme thermal resistance (see Fig. 2). One of the most common methods used since the dawn of protein engineering is the introduction of disulfide bridges,^113,114^ which usually provide considerable stability to proteins by locking their fold in a well-defined local or global conformation.^115,116^ Other common strategies involve the introduction of surface hydrogen bonds^117^ and salt-bridges^118^ that enhance protein stability by increasing protein rigidity and decreasing free energy. Indeed, an increased number of surface hydrogen bonds and salt bridges are often observed in thermophilic proteins, where they contribute to thermal stability. In mobile loops, the introduction of prolines^119^ provides an increase in stability by reducing the entropy of the denatured state. Further rational engineering strategies focus on the design of well-packed hydrophobic cores,^111^ which play a central role in preserving enzyme stability and conformational specificity. Finally, phylogenetic analysis is often used to guide rational design.^120^ Specifically, this kind of analysis helps in disfavoring uncommon residues for a specific position or favoring common ones (especially those found in thermophiles).

### B. Computational screening

Proteins may often acquire an improved stability via the introduction of several mutations. However, sometimes, a single severely destabilizing mutation is sufficient to totally disrupt a protein fold, even in the presence of several stabilizing ones. Therefore, high prediction accuracy is essential. Unfortunately, all the existing methods have a relatively high probability of introducing unfavorable mutations.^111,121^ Therefore, protein stabilization can more safely proceed only via the incorporation of a very limited number of predicted stabilizing mutations at each mutagenesis step. Alternatively, phylogenetic libraries can be used to identify optimal combinations of stabilizing mutations.^122–124^ However, both these approaches are labor-intensive and applicable only to those proteins for which well-established medium-to-high throughput screens are available. Recent methods include computational high-throughput screening methods to evaluate libraries of potentially stabilizing mutations.^125,126^

### C. Notable achievements

The earliest notable example of rational design for enzyme thermostabilization is the work of Baker and coworkers in 2005,^127^ in which they used Rosetta to stabilize a cytosine deaminase, an enzyme with potential use for antitumoral strategies. In this work, an energy function was used to evaluate target sequences threaded onto a fixed backbone. Using an iterative heuristic procedure followed by an energy evaluation step, a comprehensive search of the space sequence was done. While the adoption of those sequences associated with the lower energy values was automatic, the higher energy sequences were assigned a probability based on the Rosetta score. The authors identified three mutations that, when combined, produced a remarkable increase in enzyme stability. Following this seminal work, several other researchers introduced various computational approaches aimed at improving the stability of enzymes that are relevant for different industrial applications. Table II summarizes the key achievements in the field.

**TABLE II.** Notable examples of rational design of enzymes with industrial applications.

Enzyme	Application	Method	Stabilization achieved	References
Cytosine deaminase	Possible antitumoral	Rosetta	T_m_ increases up to 10 °C	Korkegian^127^
Glucose dehydrogenase	Commodity chemical biosynthesis	Structure-guided consensus analysis	T_m_ increases up to 34 °C	Vázquez-Figueroa *et al.*^128^
Proteinase K	Molecular biology	Phylogenetic analysis and machine learning design	20X half-life at 68 °C	Liao *et al.*^129^
Cocaine esterase	Pharmaceutical industry	Molecular d and Rosetta	30X half-life at 37 °C	Gao *et al.*^130^
Xylanase	Production of paper	Flexible region stabilization and Rosetta	15X half-life at 50 °C	Joo *et al.*^131^
Lipase	Detergents, food, bioenergy, and pharmaceuticals	B-factor analysis and Rosetta	T_m_ increases by 2 °C	Kim *et al.*^132^
Terpene synthase	Production of terpenoids	Statistical, computationally assisted design strategy (SCADS) algorithm	T_m_ increases up to 40 °C	Diaz *et al.*^133^
Lipase	Detergents, food, bioenergy, and pharmaceuticals	Consensus analysis	2X half-life in organic solvents	Park *et al.*^134^
Lipase	Detergents, food, bioenergy, and pharmaceuticals	Disulfide by Design code	T_m_ increases by 7 °C	Yu *et al.*^135^
Methyl parathion hydrolase	Detoxification of pesticides	Unfolding free energy (Prethermut code)	T_m_ increases by 12 °C, and T_50_ increases by 10 °C	Tian *et al.*^136^
Pullulanase (α-amylase)	Production of high-glucose syrup	Structure-guided consensus analysis	4.3X half-life at 60 °C	Duan *et al.*^137^
Limonene epoxide hydrolase	Production of chiral building blocks	Rosetta, Dynamic Disulfide Discovery, and Molecular Dynamics	T_m_ increases up to 35 °C and increased activity	Wijma *et al.*^126^
Cellulase	Cellulose degradation	Salt bridge design	T_m_ increases by 16 °C	Lee *et al.*^138^
3-dehydroshikimate dehydratase	Commodity chemical biosynthesis	Visual inspection, Rosetta, and void identification and packing (VIP) server	10X higher half-life at 37 °C and increased expression	Harrington *et al.*^139^
Phytase	Phytate degradation	Disulfide by Design code	3X higher half-life at 60 °C	Tan *et al.*^140^
Cutinase	Polymer degradation	Rosetta	T_m_ increases by 6 °C, 10X higher half-life at 60 °C	Shirke *et al.*^141^
Acetylcholinesterase	Detoxification of pesticides and nerve agents	Rosetta and Phylogenetic analysis	T_m_ increases by 20 °C and increased expression	Goldenzweig *et al.*^125^
Transketolase	Synthesis of complex carbohydrates	Rosetta and consensus analysis	T_m_ increases by 5 °C and increased k_cat_	Yu *et al.*^142^

### D. Rational enzyme design algorithms

In the past few years, several procedures have been developed to design thermostable enzymes. Two of them appear to be particularly promising given their general applicability, integration of orthogonal methods, and ease of use.

### E. FRESCO

The “Framework for Rapid Enzyme Stabilization by Computational libraries” (FRESCO),^126^ developed by the Janssen group at the University of Groningen, follows five major steps. In the first step (1), a library of single point mutations is generated using Rosetta, FoldX, and an in-house code for disulfide discovery, eventually excluding the catalytic region from mutable residues. The mutations are retained in the library if they provide a stabilizing effect based on internal scoring. The second step (2) involves the elimination of unreasonable mutations upon visual inspection. The major reasons for elimination are hydrophobic side chains exposed to solvent or proline residues introduced inside an α-helix. The third step (3) involves the screening of the mutant library with molecular dynamics (MD) simulations and then filtering out mutations that lead to an increase in root mean square fluctuations. The assumption for this step is that mutations that increase structural flexibility with respect to the wild type are likely to be destabilizing. After the MD-based screening, in step (4), surviving mutations are experimentally tested to verify that they provide an actual increase in melting temperature while preserving catalytic activity. At this stage, a library of experimentally validated stabilizing single-point mutations is obtained. In step (5), the validated single-point mutations are combined, screened with MD simulations and then experimentally validated, providing the final stabilized variant(s) of the enzyme.

The two major advantages of FRESCO are the use of different strategies and methods to generate the initial library of mutations and the use of orthogonal methods to filter them (i.e., Rosetta/FoldX scoring followed by MD). On the other hand, usability and automation are limited, given that step (2) involves visual inspection. A second possible drawback is that the initial stage includes the screening of only single-point mutations. Indeed, it is possible that two single-point mutations, when considered alone, are found to be destabilizing, whereas, if combined, they could generate stabilizing interactions.

### F. PROSS

A second promising framework for enzyme stabilization is the “Protein Repair One Stop Shop” (PROSS)^125^ developed by the Fleishman Lab at Weizmann Institute of Science. In the first step (1), the algorithm performs a sequence alignment with homologous proteins. Rather than selecting the most promising mutations for each position, this step is performed to eliminate the mutations that are rare or not observed. The second step (2) involves the use of Rosetta to evaluate potentially stabilizing mutations, selecting only those that provide an energy decrease with respect to the wild type. Finally, in the third step (3), the single point mutations that have been selected in the previous step are combined and the enzyme variants are ranked based on their energy score.

A major advantage of PROSS is its ease of use, either reproducing the algorithm in-house or employing the web-server developed by the authors (http://pross.weizmann.ac.il). On the other hand, a possible major limitation of this approach is the lack of orthogonal methods (e.g., MD) in combination with Rosetta. A further limitation is the need for several homologs of the target protein in order to increase the reliability of the first step of the algorithm.

### G. Outlook for rational enzyme engineering

**FIG. 3.**
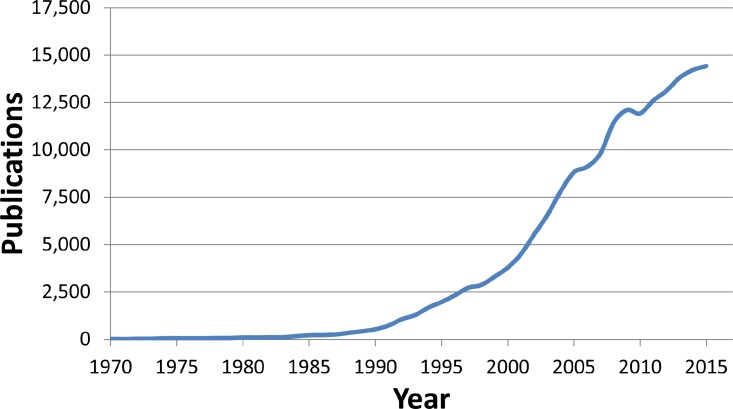
Number of publications per year on the topic of “protein design” (source: Scopus).

In the last 60 years, protein science has moved from the first success in protein structure determination (the Myoglobin X-ray crystal structure in 1958)^143^ to the early attempts at protein engineering (the design of a reduced ribonuclease in 1979).^144^ The introduction of computational analysis methods led to the *de novo* design of a protein motif in 1997,^145^ boosting our ability to engineer proteins (see Fig. 3). The possibility of building new proteins, tuning enzyme catalytic activity, and extending their thermal stability is now increasingly exploited for different industrial applications. Protein design is often termed “the inverse folding problem” because when our ability to build or modify a protein in a predictable way will finally be attained, which will provide an indirect evidence that the protein folding problem is well-understood. Therefore, the methods and successes of enzyme engineering contribute directly to those of protein structure prediction.

A general major limitation of current computational enzyme design approaches is the lack of an objective assessment of the different available methods, similar to the one used in the Critical Assessment of Structure Prediction (CASP) competition.^146^ Within the CASP challenge, research groups have the opportunity to test their structure prediction methods by identifying a protein structure from its amino acid sequence. The assessors are not part of the competitors, thus enabling objective analysis of achievements and challenges in a comparative manner. Without a similar community-wide objective assessment, the comparative analysis of computational design methods necessarily relies upon reports by the respective authors of each method, thus hampering the identification of advantages and disadvantages of each tool and the cross-dissemination of knowledge.

A further major challenge in the field of enzyme engineering is the accessibility to the general scientific community. Until now, computational enzyme design lacked standardization and solid reliability of results. As such, it has not been carried out by a large community, but rather it has clustered in a small number of labs leading the field. Most often, these are the laboratories where software packages are developed. As with other fields, it is expected that with time, more and more scientists will apply computational enzyme design, and rational design will become a standardized and common procedure carried out on a routine basis in biochemical laboratories.
